# Statistical Tests for Associations between Two Directed Acyclic Graphs

**DOI:** 10.1371/journal.pone.0010996

**Published:** 2010-06-16

**Authors:** Robert Hoehndorf, Axel-Cyrille Ngonga Ngomo, Michael Dannemann, Janet Kelso

**Affiliations:** 1 Research Group Ontologies in Medicine, Institute for Medical Informatics, Statistics and Epidemiology, University of Leipzig, Leipzig, Germany; 2 Department of Computer Science, University of Leipzig, Leipzig, Germany; 3 Department of Evolutionary Genetics, Max Planck Institute for Evolutionary Anthropology, Leipzig, Germany; 4 European Bioinformatics Institute, Hinxton, Cambridge, United Kingdom; University of East Piedmont, Italy

## Abstract

Biological data, and particularly annotation data, are increasingly being represented in directed acyclic graphs (DAGs). However, while relevant biological information is implicit in the links between multiple domains, annotations from these different domains are usually represented in distinct, unconnected DAGs, making links between the domains represented difficult to determine. We develop a novel family of general statistical tests for the discovery of strong associations between two directed acyclic graphs. Our method takes the topology of the input graphs and the specificity and relevance of associations between nodes into consideration. We apply our method to the extraction of associations between biomedical ontologies in an extensive use-case. Through a manual and an automatic evaluation, we show that our tests discover biologically relevant relations. The suite of statistical tests we develop for this purpose is implemented and freely available for download.

## Introduction

An increasing number of discoveries, particularly in biomedicine, are facilitated by statistical analyses of data annotated to biomedical ontologies [1]. Biomedical ontologies are generally represented as DAGs, and specific domains are usually represented in distinct, separate DAGs [2]–[4].

Statistical tests that utilize a single graph can only consider the given domain. However, entities from different domain are linked via biomedical relations [5]. These relations can be vital for the discovery of novel biomedical knowledge. We have designed a family of novel statistical tests to identify strong associations between nodes from two directed acyclic graphs. The tests combine measures of relevance and specificity.

We evaluated our statistical method through an extensive use-case in which we applied our tests to the detection of strong semantic associations between the Gene Ontology [3] and the Celltype Ontology [6] based on co-occurrence in scientific literature. In this use-case, we annotated the ontologies with occurrence and co-occurrence count data of the ontologies category labels in full text scientific articles. The strongest associations identified through our tests are biologically relevant relations.

An implementation of the six novel statistical tests to identify associations between directed acyclic graphs is available as free software from our project webpage at http://bioonto.de/pmwiki.php/Main/ExtractingBiologicalRelations.

### State of the art

Our approach to the computation of the strength of the association between two graphs relies on approaches for capturing the semantic similarity between categories in ontologies and for propagating these similarities within DAGs. In the following, we give a brief overview of methods for computing the similarity of categories (a more complete overview can be found in [7]). Most of the existing semantic similarity approaches assume that ontologies contain categories 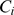 that are annotated with terms 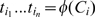. Based on this assumption, the computation of the semantic similarity of two categories 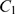 and 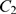 can be carried out by using the structure of the ontology to which 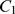 and 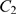 belong (edge-based approaches), the nodes and their properties (e.g., similarity between 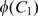 and 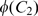) (node-based approaches) or by combining structural knowledge and annotations (hybrid approaches).

The most common edge-based approach consist of using a function of the number of edges between 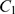 and 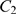 as semantic similarity measure [8], [9]. Other approaches combine the previous approach with the lenght of the path from the most specific common ancestor of 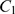 and 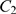 and the root node [10], [11]. Edge-based approaches rely on the nodes being elements of the same graph. Thus, they cannot be utilized when trying to compute the similarity of two nodes from distinct DAGs.

The second category of approaches, the node-based approaches, use the properties of the nodes themselves to compute their similarity. One of the central concept for using annotations to compute similarity is that of information content, which is the negative log-likehood 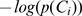 of a term 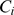 where 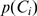 is the probability of occurrence of the terms in 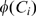 in a certain corpus. Based on this value, several similarity metrics have been developed including the information content of the most informative common ancestor used in [12], [13] or of the disjoint common ancestors [14].

In recent years, hybrid similarity measures that combine node- and edge-based approaches have been developed. Most of these approaches utilize the information content. For example [15] utilize a combination of edge weights based on node depth and node link density and of the difference of information content of the nodes linked by that edge. Other approaches such as that described in [16] compute edge weights by using a scheme that takes the type of the edge into consideration. The semantic similarity between two terms is set to a function of the maximum of the product of best path between the terms. Again, these approaches can only compute the similarity of terms from the same DAG.

The aim of our approach is to provide a means for the computation of the association between nodes from 2 DAGs, which are, in general, distinct. We do not make similar assumptions about the annotation of edges and nodes as other approaches to semantic similarity. Instead, we go beyong current semantic similarity measures by providing a measure of statistical significance in a distribution of arbitrary node and edge annotations. When applying out method to semantic similarity between ontologies, we can compute initial semantic similarity values for categories which do not belong to the same ontologies.

## Methods

### Statistics on graphs

#### Preliminaries of directed acyclic graphs

Our tests take as input two directed acyclic graphs, 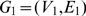 and 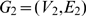 that are disjoint (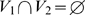). From these two graphs, a graph 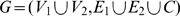 with 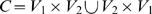 is constructed. We denote an edge as an ordered pair of vertices. If an edge connects 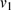 and 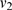, 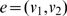, we call 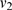 the child of 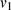 and 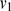 the parent of 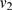. If there is a path from 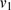 to 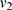, we call 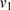 a predecessor of 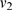 and 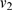 a successor of 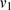.

In addition to the two graphs, two functions 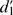 and 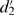 are given as input such that 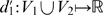 and 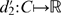. From these two functions, a graph decoration for 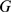 is constructed based on the assumption that the two input functions are transitive over the DAG: the decoration 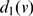 of a vertex 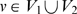 is the union of 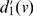 and the values of 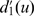 for all successors 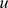 of 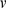. Similarly, the decoration 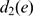 of an edge 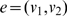 for 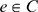 is the union of 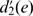 and the values of 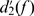 for all edges 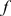 between the successors of 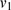 and 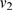.

The third component of the input is a score function 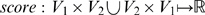. We assume that the value of the score function between the vertices 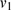 and 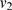 depends only on the graph decorations 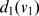 of 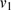 and 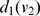 of 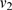 as well as the decoration 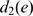 of the edge 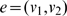.

**Figure 1:**
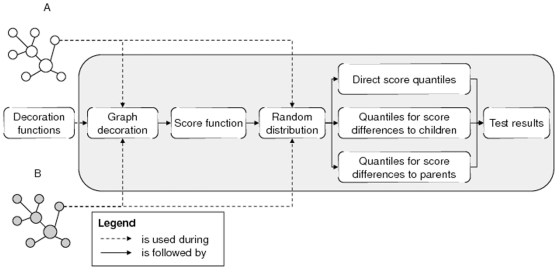
Schematic representation of our method.

The score function is not symmetric, i.e., it is not necessary that 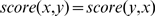. It is intended to measure the association strength between two vertices from the input graphs. Our method identifies whether the score between two vertices is significantly high. A graphical overview of our test method is shown in Figure 1.

#### Determining the Random Distribution

The score between two vertices 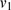 and 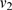 is influenced by the topology of the input DAGs: a vertex 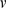 that is more general has a larger decoration set 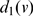 due to our basic assumption about transitivity of input graph decorations. Similarily, the cardinality of the decoration set of the edges between nodes from the two input DAGs is larger when the edges connect more general vertices. Therefore, it is insufficient to test for a high score between vertices to consider the score between two vertices as significantly high. A random distribution of the scores of each pair of vertices 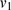 and 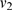 provides a means for determining the significance of the score between 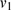 and 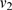. This random distribution depends on the functions 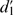 and 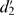, the score function and the topology of the input graphs. Hence, we cannot assume any statistical distribution of scores *ab initio*. Instead, we simulate the random distribution of the scores between each vertex pair through multiple random permutations: the 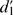-values that are given as input for our method are randomly swapped with the 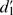-values of vertices in the input DAG from which they originate. There are two options for permutating the 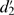-values for edges: either they are, *mutatis mutandis*, permutated similarily to the 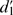-values of the vertices, or they are permutated depending on the permutation of 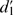-values; in the latter case, when the 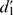-values of 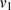 and 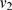 are swapped, so are the values of 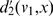 and 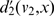 for any vertex 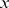.

Because our test is intended to identify associations between vertices, we do not assume that the values of 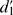 and 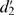 are independent. We therefore prefer to use the second option, i.e., that the permutation of the 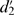 values depends on the permutation of the 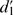-values.

Based on these permutations, we first rebuild the graph decorations 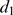 and 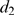. Then, we calculate and record the values of the score function 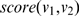 for all pairs of vertices 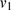 and 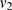. In addition, for each vertex 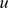, such that 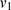 is a direct successor of 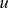, we calculate and record the score difference 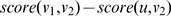. Further, for each vertex 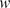 with the direct predecessor 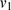, we calculate and record the difference 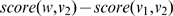.

Hence, the results of this step are threefold. First, we approximate the random score distribution for each pair of vertices through multiple random permutations. Second, each triple of vertices 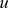, 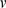 and 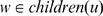 gives rise to a random distribution of score differences between 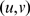 and 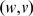. Third, each triple 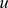, 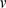 and 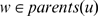 yields a random distribution of score differences between 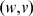 and 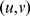.

### Ontologies as graphs

While the tests we develop can be applied to any DAG that satisfies the conditions specified above, their primary application is to test the significance of an association between categories from two ontologies. An ontology is the specification of a conceptualization of a domain [17], [18]. Many biological ontologies are represented as directed acyclic graphs (DAGs) and are available in the OBO flatfile format [2]. In these DAGs, nodes represent *categories* and edges represent *relations* between these categories. A category, also called *kind*, *class* or *universal*, is an entity that is general in reality. Examples are *dog*, *apoptosis* or *red*. Categories may have instances, of which some may not be further instantiated. These are called *individuals*. We call the set of all categories in an ontology 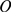 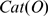.

Categories may be related to other categories. The most important relation between two categories 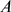 and 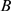 is the 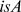 relation, 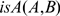. The relation 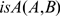 can be defined by using the instantiation relation: when 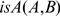, then all instances 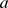 of 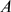 are instances of 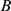 [18]. This definition implies that the 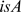 relation is reflexive, transitive and antisymmetric.

A set of categories with the 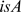 relation among them form a taxonomy. These taxonomies are often the backbone of the OBO ontologies' DAG structure. We call the set of all successors of a category 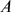 the sub-categories 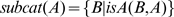 and its predecessors the super-categories 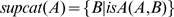. The direct successors of 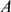 in the taxonomy are called children (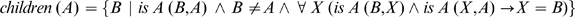), while the direct predecessors are called parents.

In the OBO flatfile format, ontologies are assigned a namespace. Category identifiers are prefixed with the namespace of the ontology to which they belong. Identifiers are therefore unique within the OBO ontologies. In addition to a unique identifier, categories are assigned a *name* and a set of *synonyms*. Neither the name nor the set of synonyms must be unique.

## Results

### Statistics on graphs

To identify strong associations, we designed a family of tests for the score of each edge between the two input DAGs that considers a fragment of the path in the DAG. The tests are designed to measure the significance of the score between vertices 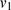 and 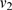 based on three criteria: (1) the score 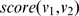 for the association should be higher than expected; (2) for each child 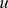 of 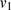, 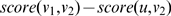 should be higher than expected; and (3) for each parent 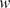 of 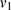, 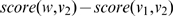 should be lower than expected.

The first criterion of our tests identifies hypothetical associations between nodes from two graphs. The second and third criteria are used to verify whether the pair is the best selection, or whether a more specific or more general association is preferable. For this purpose, the second and third criteria test for novelty of the association (compared to the child and parent nodes).

Within this section, let 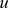 and 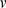 be fixed vertices from the DAGs 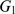 and 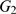, respectively. Furthermore, let 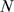 be the number of permutations that were used to determine the random distributions. The first test we designed, 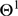, depends on the vertices 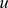 and 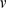, the DAG structure and the number of permutations 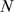. It tests for the following properties:

- the score between 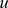 and 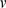 is high,
- the difference between 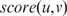 and 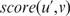 for every child 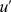 of 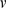 is high,
- the difference between 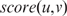 and 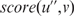 for every parent 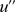 of 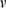 is low.

“Being high” and “being low” are captured using the values of the cumulative distribution functions (CDFs) obtained by the 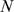 permutations performed in the previous step: one function for each pair of categories 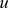 and 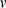, one function for each triple of categories 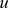, 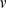 and 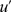 where 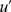 is a child of 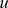, and one for each triple 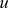, 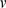 and 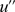 where 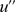 is a parent of 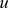. We combine the 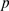-values of the score differences to children in a single value using their geometric mean. A similar combination of the score differences' 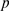-values to the parent categories of 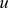 is carried out: here, the combined value is the geometric mean of 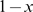, where 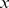 is the 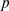-value in the corresponding CDF.

Formally, let 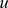 and 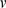 be fixed vertices from the directed acyclic graphs 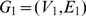 and 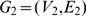, respectively, and let

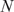
 be the number of permutations,

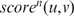
 be the score between 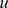 and 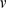 in the 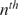 permutation,

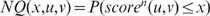
, 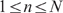, be the cumulative distribution function (CDF) of 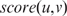.

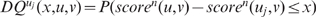
, 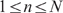, be the CDF of the difference between the vertex 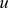 and its 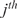 child vertex,

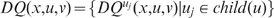
,

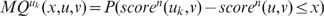
, 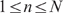, be the CDF of the score difference between the vertex 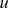 and its 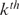 parent vertex,

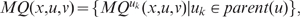
,

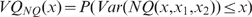
, for all 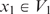 and 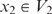, be the CDF of the variances 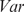 of the distribution 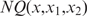, and 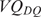 and 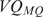 for the distributions 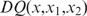 and 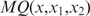, respectively.

For each child 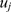 of 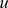, we calculate the difference in scores 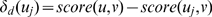. Then, we compute the geometric mean 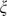 of all values 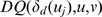. Similarly, we calculate 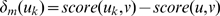 for each parent 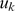 of 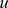, and the geometric mean 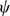 of all values 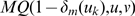. Then we define as our first test
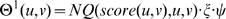
(1)


All other tests are extensions of the first test. The second test, 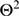, uses the minimum function instead of the geometric mean to combine the 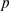-values in the CDFs of the score differences to parents and children.

The first two tests 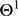 and 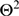 do not consider the variances of the distributions of scores, differences in scores to children and differences in scores to parents. Therefore, we extend these tests by weighting all three components of the tests with the variances of their corresponding distributions. In these tests, high variance lowers the impact of the result, while lower variance strengthens it.

We define three new distributions for the variances and choose the 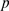-value in the respective CDF as a weight in our tests. We compute the scores for each pair of category 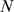 times, resulting in one distribution of scores for each pair of categories. Each of these distributions has a variance. The score variance distribution is the finite distribution (containing 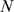 elements) of the variances of each of these distributions. We define the variance distribution for score difference to parent and child analogously.

The tests 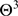 and 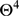 use only the variance distribution of scores, while 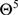 and 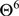 use all three variance distributions. These tests are one-sided, i.e., they are not symmetric. We define two-sided, symmetric tests 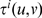 for all vertices 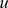 and 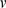 as
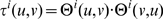
(2)
Table 1 lists the combination of properties for all tests. The precise formulation of all six tests can be found in the supplement S1.

**Table 1:** Elements of the test score of 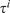.

	combining p-values in the CDF's of score differences from parents to children	variance distribution of scores	variance distributions to children and parents
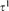	geometric mean		
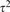	minimum		
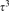	geometric mean	X	
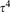	minimum	X	
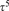	geometric mean	X	X
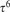	minimum	X	X

### Application to biomedical ontologies

#### Occurrence and co-occurrence count data as graph decoration

To verify whether the tests we designed yield reasonable results, we applied our method to the detection of significant co-occurrences between ontological categories in natural language texts, as a precursor to the detection of relations between ontological categories. For this purpose, we make the following assumptions:

1. A term occurs in a portion of text if it is an exact substring of this portion of text.
2. Terms can designate ontological categories; the terms that designate the same category are henceforth called the category's synset. Every occurrence of an element of the category 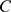
's synset is called an *occurrence of* 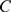. Every co-occurrence of an element of the category 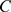
's synset with an element of the category 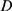
's synset is called a co-occurrence of 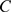 and 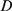.
3. If 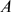 is a sub-category of 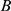, then every co-occurrence of 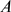 with 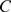 is a co-occurrence of 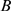 with 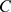. Additionally, every occurrence of 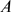 counts as an occurrence of 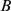.

To test our method, we used the Gene Ontology (GO) [3] and the Celltype Ontology (CL) [6] as input DAGs. The GO is an ontology specifically designed to describe gene products. It contains three separate ontologies: the biological process, molecular function and cellular component ontologies. Gene products can be tagged with ontology categories to describe and classify them. The CL is an ontology for types of cells. It classifies cells based on criteria such as structure or function.

Based on the input requirements of our test, we constructed synsets from the synonyms attached to each category in the input ontologies, and counted the occurrences and co-occurrences of the categories based on two contexts: single sentences and sentences in documents. The second context refers to whole documents, but co-occurrence is based on single sentences. Therefore, when two terms co-occur in two or more sentences within one document, their co-occurrence is only counted once. The functions that assign the occurrence and co-occurrence count values to a synset of a category for each context are called 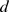 and 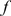, respectively.

We used exact string matching to identify terms in text. Our evaluation was conducted using a 2.2 GB text corpus containing 60143 fulltext articles from Open Access journals listed in Pubmed Central. The aim of our method is to test for significant co-occurrences between categories.

#### Text Processing

First, we counted the number of occurrences and co-occurrences of the terms contained in synsets of categories from the input ontologies. Table 2 shows examples for the synsets of categories. We counted the total number of sentences and documents in which at least one element of a synset was found by using exact matching. For each pair of categories, we counted the total number of co-occurrences of elements of their respective synsets in sentences. Furthermore, we counted the number of documents in which they co-occured within at least one sentence. We used exact matching and abstained from using any more sophisticated methods for recognizing the ontologies' categories in text [19], [20] to evaluate our method. Exact matching provides a large dataset for the evaluation of our method. For practical applications such as relationship extraction, more advanced methods should be chosen.

**Table 2:** Example synsets taken from the GO and the CL.

ID	Label	Synonyms
GO:0001574	globoside biosynthetic process	ganglioside biosynthesis; ganglioside formation; ganglioside synthesis
CL:0000114	surface ectodermal cell	cell of surface ectoderm; surface ectoderm cell

The text processing yielded, for each category 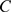, both its frequency 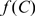 and the total number of documents in which 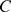 occurred, 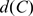. Furthermore, for each pair of categories 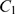 and 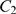, we obtained both the total number of co-occurrences in sentences 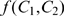 and the total number of documents containing these co-occurrences 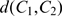.

#### Count data over ontologies

The first component in our method implements the assumption that the input graph decorations are transitive over the DAG structure. In the case of ontologies, this implements the assumption that occurrence and co-occurrence between categories is transitive over the 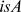 relation between categories.

We assumed that when two categories 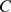 and 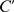 stand in the 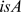 relation, 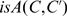, then every occurrence of 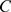 is also an occurrence of 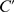. This means that the synset-closure 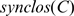 of a category 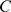 can be constructed as follows:
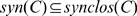
(3)

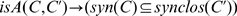
(4)For count data, the decoration value of a vertex 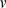 in the DAG is equal to the sum of the input value pair 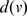 and 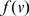 and the corresponding input values for 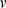
's successors. Therefore, for all categories 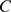, we define 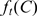 and 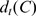 to represent the sum of the values 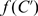 and 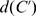 over all of 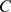
's sub-categories 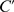. Furthermore, for all categories 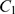 and 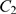, we compute the cumulated 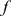 - and 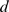-values dubbed 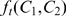 and 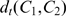:
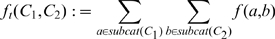
(5)

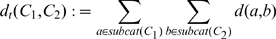
(6)Again, for count data, co-occurrence values between nodes 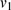 and 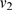 can be summed up over the successors of 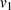 and 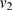 to yield the decoration of the edge between 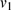 and 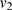.

#### A score for occurrences and co-occurrences

For all categories 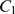 and 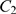, we defined the following score function:
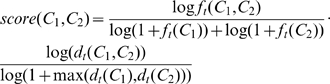
(7)The first component of the score function implements the natural logarithm of the Pointwise Mutual Information (PMI) [21] score achieved by the categories with respect to their co-occurrence within sentences. PMI has been successfully used in several text mining tools (see, e.g., [22]). To avoid divisions by 0, the denominators of all members of the score function were incremented. The second component measures a similar value using documents as context. The aim of the score function is to ensure that categories that co-occur relatively often are assigned a high score. The range of the score function is between 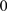 and 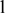.

## Discussion

### Evaluation

We applied the tests to the biological process (BP) branch of the GO and the CL. To recognize the categories in text, we used the identifier of the category, the name and all exact synonyms of the category. On average, every category had 2.1 synonyms. Using exact matching, we identified 3,751 out of BP's 14,542 (26%) categories in our text corpus. We found 491 of 754 (65%) categories from the CL. Categories from the BP co-occurred 70,967 times with CL categories.

**Figure 2:**
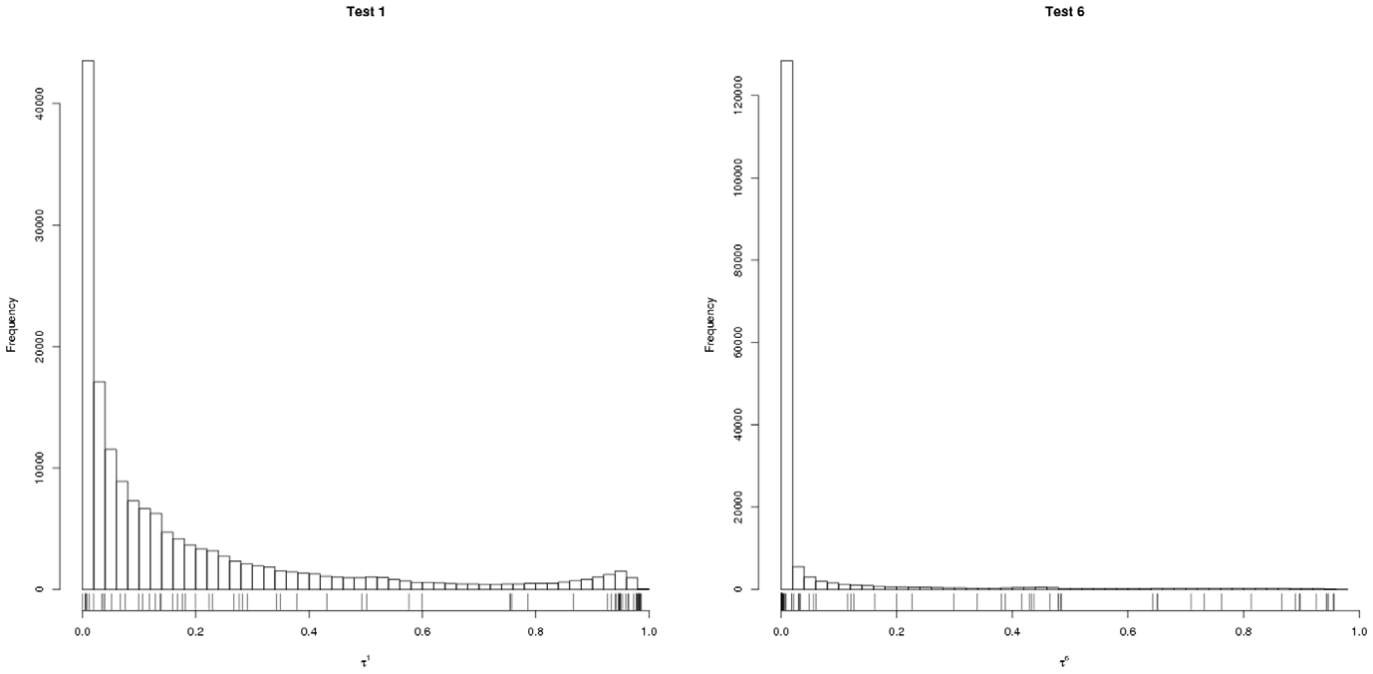
Distribution of test results. The plot on the left shows the distribution of the test results for 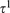. On the right, the same is shown for 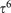. It can be seen that a test using the minimum function (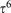) is more restrictive than a test using the geometric mean (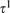). Furthermore, weighting the tests with the CDFs of the variances (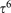) produces stronger results than the basic test (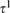). The test results of the GO-CL dataset for each test are displayed below the distributions.

Using our method, we identified a total number of 202,627 co-occurrences between categories. After applying our tests, 157,894 co-occurrences produced test values distinct from 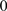. The remainder obtained a test value of 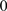 due to numerical restrictions. They were subsequently excluded, because they were indistinguishable from the absence of co-occurrence. We illustrate the quantiles obtained for different 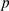-values in our six tests, 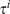, in Table 3. The distribution of scores for 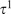 and 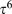 are shown in Figure 2. The remaining plots are included in the supplement S1.

**Table 3:** 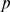
-quantiles for different 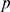-values for all tests.

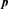 -value	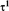	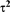	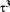	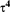	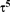	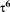
0.5	0.075	0.017	0.024	0.003	0.007	0.001
0.8	0.288	0.145	0.141	0.047	0.061	0.016
0.9	0.522	0.433	0.298	0.168	0.220	0.120
0.95	0.806	0.790	0.472	0.412	0.456	0.400
0.99	0.952	0.950	0.863	0.826	0.859	0.824

Given a 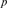-value (first column), the quantiles show the result of each test for which 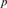-values are below the quantile.

We found that the tests using the minimum instead of the geometric mean of 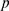-values of score differences to parent and child categories are generally more restrictive, i.e., they include fewer co-occurrences for a given cutoff. Similarly, tests including the variance for scores are generally more restrictive than tests that are not weighted by the variance of score distributions. In this sense, the tests 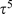 and 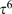 are the most restrictive.

Table 4 shows example associations, and Table 5 shows the kind of relationship between categories that our tests identified for the 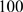 top-scoring results with respect to the test 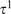. The *has-participant* relation is defined in the OBO Relationship Ontology (RO) [5] as a relation that holds between two categories, where every instance of one category participate in some instance of the other. We define the *Participates-in* relation as a relation between two categories: 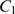 *Participates-in* 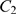 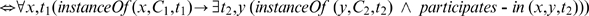, where *participates-in* is the primitive participation relation between individuals as defined in the RO. We extend the definition of *located-in* in the RO to a relation *Located-in* between processes and objects, which holds when all participants of a process are *located-in* a structure during the entire duration of the process.

**Table 4:** Association examples.

CL	GO
Myoepithelial cell	Milk ejection
Oocyte	Meiotic anaphase I
Osteoclast	Protein geranylgeranylation
Neuroblast	Neuron recognition
Keratinocyte	Keratinization
Sensory neuron	Optic nerve formation
Motor neuron	Spinal cord development
Protoplast	Photosynthesis
Lymphocyte	Chloroplast fission

The results in this table were above the quantile 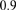 in all six tests. While the kind of relation between the categories is apparent for most results, some, like the relation between lymphocytes and chloroplast fission, remain dubious.

**Table 5:** Manually identified ontological relations in the 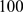 top-scoring association results with respect to 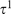.

Relation	Number of occurrences
*has-participant*	62
*Participates-in*	13
*Located-in*	2
unclassified	38

In our sample, 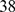 associations do not fall under one of the three relations that we investigated. We discovered several kinds of unclassified relations. First, mismatches in granularity lead to strong associations for unrelated categories. For example, *xanthine transport* and *erythrocyte* are closely related according to 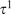. Erythrocytes are involved in the transport of xanthine. However, the GO category *xanthine transport* refers to the inter- and intracellular level of granularity, while erythrocytes transport nutrients between organs. Second, some categories are indirectly related via another category. For example, osteoclasts and lymph node development are related via the protein RANK. Third, when cells have closely related functions, we sometimes identify too specific or too generic cell types as in the case of the association between *basophil degranulation* and *mast cell*. Finally, 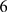 out of 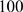 associations in our sample seem erroneous.

We were not able to compute precision or recall for our method due to the absence of a gold standard. However, we compared our method with the GO-CL crossproducts available from the OBO Foundry. The dataset contains manually verified relations between categories from the GO and the CL that have been extracted using pattern matching on category names [23]. As this method is based on the compositional nature of terms in the GO, it exclusively identifies relations in which one category name (usually a type of cell) is a substring of another category name (usually a GO category).

The GO-CL crossproduct contains 396 relations between GO and CL categories. From these 396, we identified 73 that co-occurred in our text corpus. Table 6 shows the percentage of significant co-occurrences within these 73 relations for different cutoffs in our six tests. Figure 2 shows the distribution of the 73 pairs with respect to 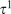 and 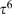.

**Table 6:** Evaluation of our approach with respect to the GO-CL dataset [23].

Recall	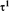	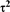	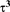	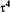	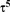	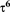
99%	0.004	0	0	0	0	0
95%	0.007	0.006	0.003	0	0.002	0
80%	0.102	0.054	0.028	0.003	0.016	0.002
70%	0.173	0.109	0.049	0.008	0.029	0.004
50%	0.502	0.350	0.173	0.063	0.154	0.060

The dataset we used for comparison consists of the 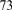 relations from the GO-CL crossproduct [23] found in our text corpus. Columns two to seven show the cutoff values required to identify the percentage given in column one of associations as significant using tests one to six. For example, at a cutoff of 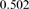, 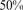 of the relations found in the dataset were significant according to test 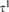.

As our method relies exclusively on the distribution of terms and not on their syntactic structure, it permits the recognition of associations between categories that cannot be recognized using syntactic patterns. An example of such an association is *myoepithelial cell* (cells located in the mammary gland) and *milk ejection*.

Important potential applications for our tests arise from the fact that annotations of a large set of biomedical ontologies satisfy the conditions for our tests. Annotations satisfy the True Path Rule [3]: if two categories 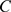 and 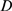 stand in the *is-a* or *part-of* relation, then any annotation of 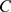 is also an annotation of 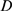. Therefore, if gene annotations are used as graph decorations for the two input graphs of our method, the conditions for applying our tests are satisfied. For detecting associations between annotations, an appropriate score function must be chosen based on the hypothesis that is to be tested.

Another potential application of our tests lies in the field of relation extraction. The evaluation of our tests with the GO and CL reveals that we are able to detect biologically relevant associations between these ontologies. 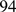 of the best 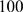 associations retrieved by 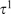 have biological meaning, as shown in Table 5. Although our approach is unable to detect the types of the biological relations, the associations provide a good starting point for an elaborate approach to the extraction of biological relations.

Our method is designed for the detection of associations between two DAGs. However, it can be generalized to test for associations between 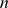 graphs. The result of the tests would then be significant 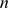-ary associations between 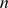 nodes from 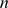 graphs.

### Conclusions

We developed a family of novel statistical tests for associations between two directed acyclic graphs. The tests account for the graphs' topologies and test for relevance and specificity of associations. The tests are suitable for the detection of associations between categories from two biomedical ontologies, in particular those which comply with the OBO criteria [24].

In an extensive use-case, we applied our tests to the discovery of associations between categories from the Gene Ontology and the Celltype Ontology that were decorated with the number of occurrences and co-occurrences of the categories' labels in a large corpus of full-text articles. Our results show that a large proportion of the associations discovered by our tests are biologically relevant relations.

The family of tests is implemented in a Java library, which is available as free software from our project webpage at http://bioonto.de/pmwiki.php/Main/ExtractingBiologicalRelations.

## Supporting Information

Supplement S1 Statistical tests for associations between two directed acyclic graphs and their application to biomedical ontologies.
(0.14 MB PDF)
