# Supplementary material for: Statistical Tests for Associations between Two Directed Acyclic Graphs
Source: PLoS One. 2010 Jun 16;5(6):e10996. doi: 10.1371/journal.pone.0010996 (PMC2886832; doi:10.1371/journal.pone.0010996)
Supplement: Supplement S1 — Statistical tests for associations between two directed acyclic graphs and their application to biomedical ontologies. (0.14 MB PDF) [file pone.0010996.s001.pdf]

Supplement: Statistical tests for associations between two directed acyclic graphs and their application to biomedical ontologies

Robert Hoehndorf[1,2,3,*], Axel-Cyrille Ngonga Ngomo[2], Michael Dannemann[3], Janet Kelso[3]
1 Research Group *Ontologies in Medicine*, Institute for Medical Informatics, Statistics and Epidemiology, University of Leipzig, Leipzig, Germany
2 Department of Computer Science, University of Leipzig, Leipzig, Germany
3 Department of Evolutionary Genetics, Max Planck Institute for Evolutionary Anthropology, Leipzig, Germany
$*$ **E-mail: leechuck@acm.org**

1 Statistical Tests

Within this section, let u and v be fixed vertices from the directed acyclic graphs $G_1 = (V_1, E_1)$ and $G_2 = (V_2, E_2)$, respectively. Furthermore, let

- N be the number of permutations,
- $score^n(u, v)$ be the score between u and v in the nth permutation,
- $NQ(x, u, v) = P(score^n(u, v) \leq x)$, $1 \leq n \leq N$, be the cumulative distribution function (CDF) of $score(u, v)$.
- $DQ^{u_j}(x, u, v) = P(score^n(u, v) - score^n(u_j, v) \leq x)$, $1 \leq n \leq N$, be the CDF of the difference between the vertex u and its jth child vertex,
- $DQ(x, u, v) = \{DQ^{u_j}(x, u, v) | u_j \in child(u)\}$,
- $MQ^{u_k}(x, u, v) = P(score^n(u_k, v) - score^n(u, v) \leq x)$, $1 \leq n \leq N$, be the CDF of the score difference between the vertex u and its kth parent vertex,
- $MQ(x, u, v) = \{MQ^{u_k}(x, u, v) | u_k \in parent(u)\}$,
- $VQ_{NQ}(x) = P(Var(NQ(x, x_1, x_2)) \leq x)$, for all $x_1 \in V_1$ and $x_2 \in V_2$, be the CDF of the variances Var of the distribution $NQ(x, x_1, x_2)$, and VQ_{DQ} and VQ_{MQ} for the distributions $DQ(x, x_1, x_2)$ and $MQ(x, x_1, x_2)$, respectively.

For each child u_j of u, we calculate the difference in scores $\delta_d(u_j) = score(u, v) - score(u_j, v)$. Then, we compute the geometric mean ξ of all values $DQ(\delta_d(u_j), u, v)$. Similarly, we calculate $\delta_m(u_k) = score(u_k, v) - score(u, v)$ for each parent u_k of u, and the geometric mean ψ of all values $MQ(1 - \delta_m(u_k), u, v)$. Then we define as our first test

$$\Theta^1(u, v) = NQ(score(u, v), u, v) \cdot \xi \cdot \psi \quad (1)$$

All other tests are extensions of the first test. The second test uses the minimum function instead of the geometric mean. Let μ be the minimum of $DQ(\delta_d(u_j), u, v)$, and ν be the minimum of $MQ(1 - \delta_m(u_k), u, v)$. Then, we define

$$\Theta^2(u, v) = NQ(score(u, v), u, v) \cdot \mu \cdot \nu \quad (2)$$

For the remaining tests, we define the CDFs VQ_{NQ}, VQ_{DQ} and VQ_{MQ} for the variance in all NQ, DQ and MQ, and consecutively use the p-values of the measured variance. Then, we define the tests Θ^3 and Θ^4 as

$$\Theta^3(u, v) = \Theta^1(u, v) \cdot VQ_{NQ}(1 - Var(NQ(x, u, v))) \quad (3)$$

and

$$\Theta^4(u, v) = \Theta^2(u, v) \cdot VQ_{NQ}(1 - Var(NQ(x, u, v))) \quad (4)$$

For the final two tests Θ^5 and Θ^6, we weight each element used in the geometric mean and minimum functions in the tests Θ^3 and Θ^4 using the variance of the corresponding distributions. For Θ^5, this means normalizing the test Θ^3 with the geometric means τ and λ of the sets $VQ_{DQ}(1 - Var(DQ(x,u,v)))$ and $VQ_{MQ}(1 - Var(MQ(x,u,v)))$:

$$\Theta^5(u,v) = \Theta^3(u,v) \cdot \lambda \cdot \tau \tag{5}$$

For the final test, we let the minimum function run over the values weighted by the variances. It is similar to test Θ^4, except that the values $\delta_d(u_j)$ and $\delta_m(u_k)$ in the computation of μ and ν are replaced by $\delta'_d(u_j) = \delta_d(u_j) \cdot (1 - VQ_{DQ}(Var(DQ^{u_j}(x,u,v))))$ and $\delta'_m(u_k) = (1 - VQ_{DQ}(Var(MQ^{u_k}(x,u,v))))$.

2 Precise formulation

Equations 7 to 12 show the precise mathematical formulation of our tests. The implementation of the six tests in Groovy is available on the project webpage. The tests illustrated in these equations are one-sided: they test the specificity of a co-occurrence based only on the ontology of the category that is used as the first argument. The final tests τ^i are the two-sided versions of the tests Θ^i presented above, and are defined as:

$$\tau^i(u,v) = \Theta^i(u,v) \cdot \Theta^i(v,u) \tag{6}$$

Let u_c^j be the jth child of the vertex u. Then, let $d^j = score(u,v) - score(u_c^j, v)$. Let u_p^i be the ith parent of the vertex u. Then, let $m^i = score(u_p^i, v) - score(u,v)$.

$$\Theta^1(u,v) = NQ(score(u,v),u,v) \cdot \left(\prod_j DQ(d^j,u,v)\right)^{1/N} \left(\prod_j \left(MQ(1-m^j,u,v)\right)\right)^{1/N} \tag{7}$$

$$\Theta^2(u,v) = NQ(score(u,v),u,v) \cdot \min\left(DQ(d^j,u,v)\right) \min\left(MQ(1-m^j,u,v)\right) \tag{8}$$

$$\Theta^3(u,v) = NQ(score(u,v),u,v) \cdot \left(\prod_j DQ(d^j,u,v)\right)^{1/N} \left(\prod_j \left(MQ(1-m^j,u,v)\right)\right)^{1/N} \cdot VQ_{NQ}(1 - Var(NQ(x,u,v))) \tag{9}$$

$$\Theta^4(u,v) = NQ(score(u,v),u,v) \cdot \min\left(DQ(d^j,u,v)\right) \min\left(MQ(1-m^j,u,v)\right) \cdot VQ_{NQ}(1 - Var(NQ(x,u,v))) \tag{10}$$

$$\begin{aligned}\Theta^5(u,v) = {} & NQ(score(u,v),u,v) \cdot \left(\prod_j DQ(d^j,u,v)\right)^{1/N} \left(\prod_j \left(MQ(1-m^j,u,v)\right)\right)^{1/N} \cdot \\ & VQ_{NQ}(1 - Var(NQ(x,u,v))) \cdot VQ_{DQ}(1 - Var(DQ^j(x,u,v))) \cdot VQ_{MQ}(1 - Var(MQ^k(x,u,v)))\end{aligned} \tag{11}$$

$$\begin{aligned}\Theta^6(u,v) = {} & NQ(score(u,v),u,v) \cdot \min\left(DQ(d^j,u,v)\right) \min\left(MQ(1-m^j,u,v)\right) \cdot \\ & VQ_{NQ}(1 - Var(NQ(x,u,v))) \cdot VQ_{DQ}(1 - Var(DQ^j(x,u,v))) \cdot VQ_{MQ}(1 - Var(MQ^k(x,u,v)))\end{aligned} \tag{12}$$

3 Distributions

In Figure 1, the remaining plots of the distributions of test results for the tests τ^2, τ^3, τ^4 and τ^5 are shown, together with their overlap with the GO-CL dataset.

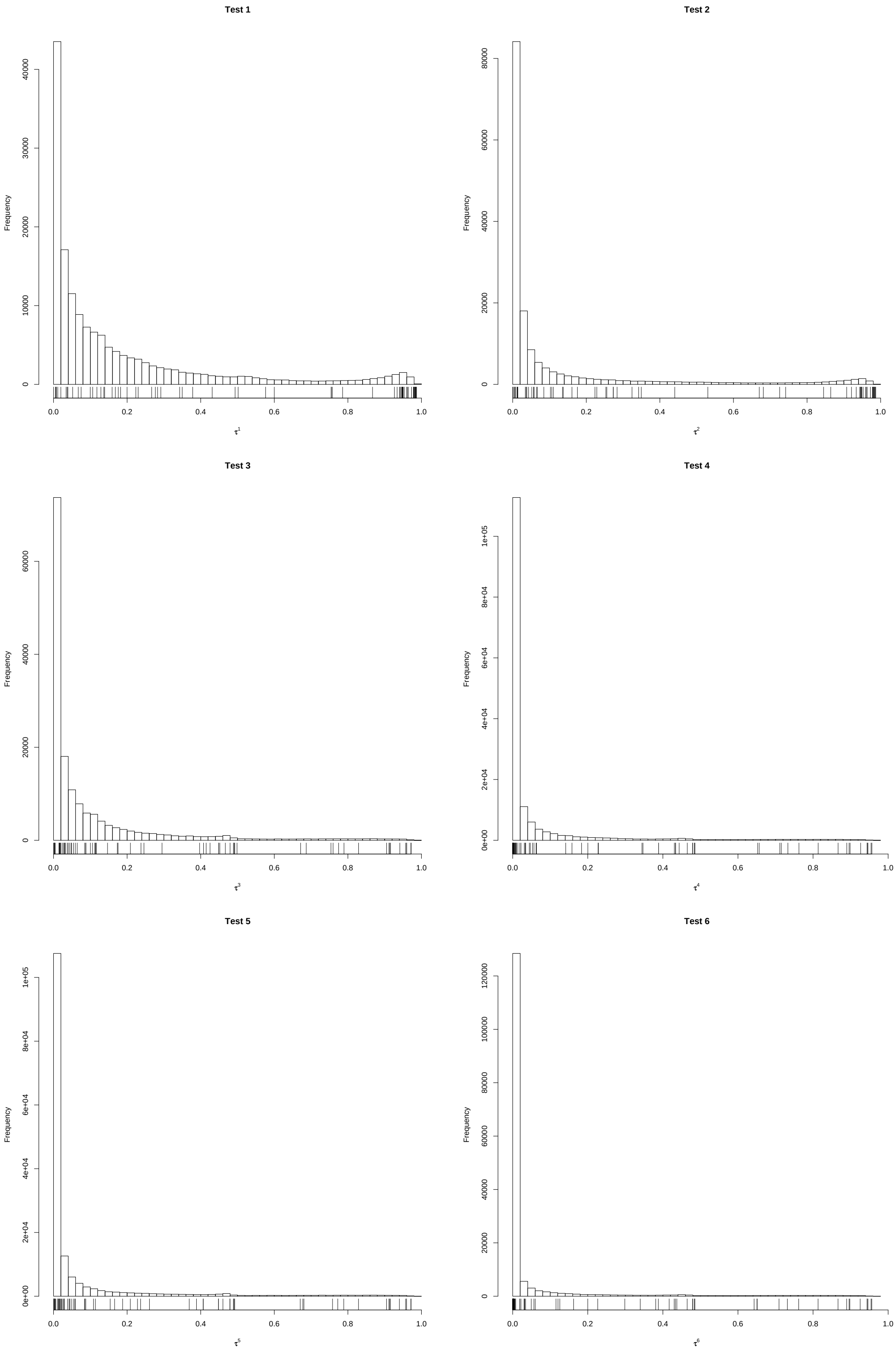

Figure 1. Distribution of test results. The plots show the distributions of the test results for all τ^i. Below the distributions, the quantiles of the GO-CL dataset for each test are displayed.
